# Buffalo Medical Library Association

**Published:** 1887-12

**Authors:** William D. Granger, John Hauenstein, F. W. Bartlett


### Communications to the Editor.

BUFFALO, N. Y., November 17, 1887.

*Editors Medical and Surgical Journal:*

It was the expressed wish, at the last meeting of the Medical Library Association, that the report of its committee, which I send you, should be published in the Buffalo Medical Journal prior to the annual meeting, December 2d. Owing to the fact that the journals do not, as a rule, appear on the first day of the month, it is hoped the meeting will be adjourned to Friday, December 9th. The place of meeting will be in the library-room of the Buffalo Medical College, and the hour of meeting, four P. M. The profession are invited to be present. Those only who have paid their dues for the year, can vote at the meeting. The dues are three dollars, and can be paid any time to Dr. Hinckle, acting-treasurer. I suppose, arrangements will be made by which payments can be made at the meeting. In case adjournment is made, some public notice will be given.

Truly yours,

WILLIAM D. GRANGER.

### *BUFFALO MEDICAL LIBRARY ASSOCIATION.*

REPORT OF A COMMITTEE UPON PROPOSALS TO MERGE THE LIBRARY WITH THE BUFFALO MEDICAL COLLEGE LIBRARY, OR WITH THE BUFFALO (YOUNG MEN'S) LIBRARY.

At a meeting of the Directors of the Buffalo Medical Library Association, held October 28, 1887, the undersigned were appointed a committee to consider propositions made by parties willing, under certain conditions, to assume the care of our Library. We were also directed to report at the annual meeting, held on the first Friday in December next.

We, therefore, make the following report:

In order to fully understand the present condition of the Library, we would briefly review the story of its history and its struggles. The Library is now ending its fifth year. It has kept its obligation with the profession, but finds itself without professional support. Every demand worthy of consideration, to make the Library more popular, has been adopted. We still keep upon our tables a fresh supply of the best medical periodicals of this country and Europe; we have made the periodicals available for home use almost as soon as received; we have daily kept the Library open for consultation, and provided for some one to be present to attend to visitors, and we have reduced the annual dues to three dollars a year.

We have the foundation for a good library. We have over five years of subscriptions of periodicals, mostly bound; we have over 150 bound books; we have valuable gifts of pamphlets, files of journals, etc., ready to be bound. As an example, we may mention an almost complete file of the *American Journal of Medical Science.* In a word, the Library is just getting into a position to be useful, and command the respect of the profession, its support and its gifts.

The location of the Library has, from the first, been the most important matter for the directors to decide upon. It was debated at length, in the meeting for organization, by the profession at large, and, undecided by them at that time, has been the subject of their discussion at each annual meeting. The directors have tried to secure a central location, to have the rooms open daily, and some one present to attend to visitors. In these efforts, they have been successful.

At first, the Library was in a private house, but the rent—$200 a year—made it necessary to remove to some less-expensive place. After much deliberation, it was unanimously decided, in 1885, to accept the offer of the Buffalo Medical College, and the Library was removed there, where it still remains. We pay them fifty-two dollars a year. In return, they keep the library-room open six hours a day, and have some one present to attend to visitors. They also attend to all details for the protection of our property. The other expenses of the Library, including about eighty dollars for journals, and something for incidentals, make the expenditures nearly $150 dollars a year.

This small sum cannot be raised by the profession, and the end of each year finds us in debt. Again and again, the friends of the Library have paid its debts. But the utter want of interest by the profession gives them little encouragement to continue this practice. It is strange that, as the Library becomes more valuable, its support becomes less and less.

The following show the amounts of annual dues received from the organization of the Library:

| | |
|---|---|
| 1883 | $ 305 |
| 1884 | 255 |
| 1885 | 145 |
| 1886 | 113 |
| 1887 | 74 |
| Total | $ 892 |
| Individual subscriptions | 170 |
| Total | $1,062 |

Would that the profession still had the desire to support the Library with $300 a year, and continue to do so for five years! Then, with its diminished expenses, it would grow rich in all that is desirable for a library to be, and, instead of a shame, be a source of pride to our profession, and become too valuable to be neglected.

The Library, however, ends the year with a greatly-diminished income, a debt of over $100, and the yearly subscriptions of our journals expiring.

It seems impossible to continue our present modest expenses, and your committee feel obliged to advise you to accept one of the two offers made us by those willing to care for our Library, to give it, without cost, shelf-room.

The first offer to consider is that of the Buffalo Medical College. They offer to take in trust our Library, subject to our recall at any time. They agree to keep up our files of journals; the additions they pay for, to belong to the College. They will make the Library a free consulting Library for the whole profession; the right to a circulating use of the Library to be obtained by paying a fee of not more than three dollars a year.

We would suggest, if the above propositions be agreed to, that a full list of property turned over be made, signed by representatives of Library and College; the College to be responsible for the safe-keeping of our property without any expense on our part; that they yearly make a report to the Library Association, at its annual meeting, of the condition of our property; that we reserve the right to keep up any of our files, and that all books, pamphlets and periodicals added by us shall belong to our Library Association, and be cared for without cost, and returned to us on demand; that a committee of three, representing the Library, shall have access, at any reasonable time, to inspect the condition of our Library, and that a committee of three be appointed to arrange for, and make the necessary transfer.

The Buffalo Library Association is willing to receive our books, bound pamphlets and periodicals, and such other material as can be placed on shelves, and keep them apart from the general library as a distinct collection. Our periodicals, which they wish us to continue, will be placed in their general reading-room. Members of their association are to have, under certain restrictions, the use of our property, and members of the Medical Library Association are also to enjoy the right of use of our Library, though not members of the Buffalo Library Association.

We would suggest, if these propositions are accepted, to insist upon the same safeguards for the protection of our property as we suggested in the case of the Medical College.

The committee do not advise which of the above propositions should be accepted. But we do advise that it is necessary to accept one of them, if the property we now own is to be cared for, and our Association saved from ruin.

William D. Granger.
John Hauenstein.
F. W. Bartlett.

